# Molecular characterization, expression of chicken TBK1 gene and its effect on IRF3 signaling pathway

**DOI:** 10.1371/journal.pone.0177608

**Published:** 2017-05-11

**Authors:** Yan Wang, Yue Yin, Xi Lan, Fei Ye, Kai Tian, Xiaoling Zhao, Huadong Yin, Diyan Li, Hengyong Xu, Yiping Liu, Qing Zhu

**Affiliations:** 1 Farm Animal Genetic Resources Exploration and Innovation Key Laboratory of Sichuan Province, Sichuan Agricultural University, Chengdu, China; 2 Institute of Laboratory Animal of Sichuan Academy of Medical Sciences & Sichuan Provincial People’s Hospital, Chengdu, China; University of Hong Kong, HONG KONG

## Abstract

TRAF family member-associated NF-κB activator (TANK)-binding kinase1 (TBK1) is a serine-threonine kinase at the crossroads of multiple interferon (IFN)-inducing signaling pathways in innate immunity. The importance of TBK1 in antiviral immunity is well established in mammal models, but in chicken, the molecular characterization and potential function of TBK1 remain unclear. In the present study, the open-reading frame (ORF) of chicken *TBK1* (*chTBK1*) was cloned and characterized. The sequencing results revealed that the *chTBK1* ORF consists of 2190 base pairs (bp) encoding a deduced protein of 729 amino acid residues. Multiple sequence alignment analysis demonstrated chTBK1 similarity to other birds and mammals, which indicates that it is evolutionarily conserved. Quantitative real-time PCR (qRT-PCR) results showed that *chTBK1* was ubiquitously expressed in chicken tissues and expression was especially high in immune tissues. In addition, the expression of *chTBK1* was significantly up-regulated by infection with avian leukosis virus subgroup J (ALV-J) both *in vivo* and in chicken embryo fibroblasts (CEFs) challenged with ALV-J or stimulated with poly I:C *in vitro*. Consistent with the activation of *chTBK1*, the interferon regulatory factor 3 (IRF3) and IFNβ gene in CEFs were also up-regulated after challenge with ALV-J or polyI:C. In contrast, the expression *of IRF3* and *IFNβ* in CEFs was significantly reduced by siRNA targeting the *chTBK1* gene compared with a negative control (NC) during ALV-J infection or polyI:C transfection. In conclusion, our results demonstrated that chTBK1 may be an important immunoregulator for IRF3 and IFNβ induction in response to viral stimulation in chicken.

## Introduction

TBK1, also termed NF-κB activating kinase or TRAF2-associated kinase, was identified as an important adaptor molecule linking upstream receptor signals to downstream gene activation in apoptotic, inflammatory and immune responses [1,2]. TBK1 is a serine-threonine kinase, which belongs to the inhibitor of κB kinase (IKK) family [3]. As a non-canonical IKK family member, TBK1 possesses 61% sequence identity with IKKε (another non-canonical IKK member) and shares 27% primary sequence identity with IKKα/β (the canonical IKK members) [4]. Due to the sequence similarity, TBK1 is structurally identical to the other IKKs proteins, which present a trimodular architecture with an N-terminal kinase domain, an ubiquitin-like domain (absent in IKKα) and an α-helical scaffold/dimerization domain [5]. The kinase domain of TBK1, which contains the catalytic activity, is highly conserved [6]. The ubiquitin-like domain, which is adjacent to the kinase domain, is a regulatory component of the TBK1 kinase that positively controls TBK1 kinase activation and signal transduction [7]. The scaffold/dimerization domain, which extensively interacts with the kinase domain and the ubiquitin-like domain, contributes to the dimerization and substrate recognition of TBK1 [5].

In innate immunity, TBK1 was proposed to be a hub protein, which has been implicated in various pattern recognition receptors (PRRs). For instance, in the endosomal membrane, Toll-like receptor 4 (TLR4) and TLR3 recognized bacterial endotoxin or lipopolysaccharide (LPS) and virus or double-stranded RNA (dsRNA), respectively. Both TLR4 and TLR3 activated TBK1 via the recruitment of the adaptor protein called Toll/IL-1 receptor domain-containing adaptor-inducing IFNβ (TRIF) and tumor necrosis factor receptor-associated factor 3 (TRAF3) [8]. In the cytoplasm, the presence of virus particles or dsRNA initiated immune responses via retinoic acid-inducible gene I (RIG-1)-like receptors (RLRs), such as RIG-1 and melanoma differentiation-associated gene 5 (MDA-5), which assemble another adaptor molecule termed mitochondrion-associated adapter IFNβ promoter stimulator 1 (MAVS) as well as TRAF3 to evoke TBK1 [9,10]. Recently, an intracellular adaptor protein stimulator of interferon genes called STING, which was activated by cytoplasmic DNA receptors, also funneled into TBK1-specific activation [11, 12].

Initial studies showed that TBK1 possesses the ability to induce IκB degradation and NF-κB activity via TANK and IKKβ [13,14]. However, the role of TBK1 in NF-κB signaling pathways is controversial. TBK1 null fibroblasts exhibited normal NF-κB activation in response to tumor necrosis factor α (TNFα), interleukin-1 and virus infection [15]. Interestingly, following those discoveries, TBK1 and IKKε, a close homolog of TBK1, were confirmed as important kinases for IFN signaling induction [16]. Although both of the kinases have been found to involve IRF3/7 and they both share the common upstream adaptors, TBK1 and IKKε have largely distinct functions. TBK1 plays an indispensable role in IFN induction because the activation of IRF3 and IFNβ is impaired in TBK1-defective (not IKKε-defective) murine fibroblasts after virus infection [17]. Later, two important observations showed that TBK1 mainly functioned on ubiquitously expressed IRF3 to induce type I interferon activation, whereas IKKε preferred IRF7 and is required for the activation of IFN-stimulated genes by STAT1 [18,19]. While TBK1 was found to be constitutively expressed in many cells, IKKε was found to be primarily expressed in the cells of lymphoid tissues [18]. All of these results indicated that TBK1 functions as a key node protein to induce an early phase of IFN responses after challenge by viruses and bacteria.

TBK1 has been well-characterized in mammals, but few studies have investigated the sequence, tissues distribution and its possible function in chickens. To investigate these questions, we cloned and characterized the full-length coding sequence of *chTBK1* and examined the tissue-specific expression of *chTBK1* mRNA. Moreover, we detected *chTBK1* expression differences both in *vivo* and in *vitro* in chickens infected with ALV-J and in CEFs transfected with polyI:C, respectively. In addition, we measured the relative expression of *IRF3* and *IFNβ* in CEFs stimulated with ALV-J or polyI:C. Furthermore, we examined the role of *chTBK1* in TBK1-impaired CEFs challenged with ALV-J and polyI:C.

## Methods

### Ethics statement

The animal studies and sample collection were conducted according to the guidelines approved by the Sichuan Agricultural University Institutional Animal Care and Use Committee in College of Animal Science and Technology, Sichuan Agricultural University, Sichuan, China (DKY-B201000805).

### Cloning and sequencing of chicken TBK1

Based on the published reference sequence of *TBK1* from *Gallus gallus* (GenBank accession number: NM_001199558.1), a pairs of gene specific primers (Table 1) for the amplification of the complete coding region were designed using Primer Premier 5 software (Premier BioSoft, Palo Alto, CA, USA). Spleen complementary DNA was used as the template for amplification. Polymerase chain reaction (PCR) was carried out with the initial denaturation at 94°C for 5 min and 34 cycles of 94°C for 45 sec, 60°C for 30 sec and 72°C for 1 min. A final elongation step was conducted at 72°C for 10 min. The PCR fragments were purified by the Gel Extraction Mini Kit (Qiagen, Germany) and were cloned into the pMD-19T vector (Takara, Japan). The plasmid DNA was isolated using a Plasmid Mini Kit (Qiagen, Germany) and was then sequenced by BGI Biotechnology Company (Beijing, China).

**Table 1:** Gene-specific primers used for clone and RT-PCR analyses in this study.

Purpose	Primer	Sequence (5’-3’)	Product size/bp	AT
Clone	chTBK1-F	CTCGAGATGCAGAGCACCTCGAATT	2200	60
	chTBK1-R	GGATCCGAGATACAGTCCACATTCC		
RT-PCR	TBK1-F	GGTTTGCCAGAATCGGAGT	227	61
	TBK1-R	TGTAAATACTCCTCTGTGCCGT		
	GAPDH-F	AGGACCAGGTTGTCTCCTGT	153	57
	GAPDH-R	CCATCAAGTCCACAACACGG		
	IRF3-F	TACACTGAGGACTTGCTGGAGGT	170	62
	IRF3-R	AAGATGGTGGTCTCCTGATCC		
	IFNβ-F	CCTCAACCAGATCCAGCATTAC	167	59
	IFNβ-R	CCCAGGTACAAGCACTGTAGTT		
	Env-F	TGCGTGCGTGGTTATTATTTC	144	56.6
	Env-R	AATGGTGAGGTCGCTGACTGT		

F = forward primer, R = for reverse primer; AT-Annealing temperature (°C)

### Viruses, chicken and tissue collection

The ALV-J virus used in this study was obtained from the Veterinary Medicine of Sichuan Agricultural University (Ya’an, China) and was stored at -80°C until inoculation. All of the experiments using infectious virus were conducted in biosafety level 2 (BSL-2) facilities at the Farm Animal Genetic Resources Exploration and Innovation Key Laboratory of Sichuan Agricultural University (Ya’an, China).

Specific pathogen free (SPF) chicken eggs (White Leghorn) were purchased from Merial Company (Beijing, China). The eggs hatched in the Sichuan Agricultural University (Ya’an, China) poultry farm and were reared in filtered-air, positive-pressure isolators with free access to feed and water (Sichuan, China).

To detect the tissue distribution of *chTBK1*, three healthy chickens were euthanized at the age of 3 weeks and 11 tissue samples, including the heart, liver, spleen, lung, kidney, thymus, small intestine, brain, pectoral muscle and leg muscle of each chicken were harvested and immediately snap frozen in liquid nitrogen for total RNA extraction.

### ALV-J challenge and sample collection

On the hatch day, 120 birds were randomly divided into two groups: one group was intraperitoneally inoculated with a total ALV-J virus dose of 10^6^ TCID_50_ in 0.4 ml, and the other group was treated with an equal volume of phosphate buffered saline only (0.4 ml) as the mock control. Five chickens of each group were euthanized at 3, 5, 7, 9, 14, 24 and 42 days post-infection (dpi), and the immune tissues, including spleen, bursa of Fabricius and thymus, were collected and preserved at -80°C.

### Cell culture and treatment, RNA interference experiments

CEFs were harvested from the 9-day-old embryonated SPF chicken eggs and were cultured in Dulbecco’s minimal essential medium containing 10% fetal bovine serum (Gibco, USA), 100 U/ml penicillin, and 100 μg/ml streptomycin. The cells were seeded in 12-well plates at 10×10^4^ cells/ml and were maintained at 37°C in a 5% CO_2_/air environment. At 80%-90% confluency, the cells were washed in serum-free medium and stimulated with ALV-J or polyI: C (Invitrogen, USA). For virus challenge, the cells were incubated for 1 hour with 200 μl ALV-J virus (multiplicity of infection of 1) per well. PolyI:C was transfected into the cells using Lipofectamine 2000 (Invitrogen, USA) according to the manufacturer’s instructions. At 2, 6, 12 and 20 hours post-infection (hpi), the cells were collected in TRIzol reagent for further analysis.

A small interfering RNA (siRNA) targeting the *ch*TBK1 gene was designed and synthesized by a commercial company (Invitrogen, USA). Two siRNA sequences targeting 59–79 (siTBK1.1) and 983–1083 (siTBK1.2) of the *chTBK1* ORF were selected with the corresponding siRNA sequences of 5’-GCTGTTGTCTGACATTCTAGG-3’ (siTBK1.1) and 5’-GCAGATGACCTTGCACAAAGT-3’ (siTBK1.2), respectively. The negative control (NC) oligonucleotides were purchased from Invitrogen. SiRNA duplexes were transfected into cells using Lipofectamine 2000 according the manufacturer’s instructions. After 4 hours, the mix was removed and was replaced with DMEM with 5% FBS. At 22, 28 and 36 hours post-transfection, the cells were collected in TRIzol reagent to verify the knockdown of *chTBK1* (to detect the interference efficiency). In addition, at 24 hours post-transfection, the cells were challenged with virus and polyI:C to evaluate the influence of chTBK1 knockdown on the expression of endogenous genes.

### RNA extraction and reverse transcription

Total RNA was extracted from tissue samples or CEFs using the TRIzol reagent (Invitrogen, USA) according to the manufacturer’s instructions. The RNA was eluted in 30 μl DNase/RNase-Free water (Takaka, Japan) and was stored at -80°C for reverse transcription.

Before reverse transcription, the total RNA for integrity and quality using electrophoresis (1% agarose gels with ethidium bromide) and a Nanodrop spectrophotometer (Nanodrop 2000C, Thermo Scientific, USA), respectively. Reverse transcription was carried out using the PrimeScript RT reagent Kit with gDNA Eraser (Takaka, Japan) according to the manufacturer’s instructions.

### Quantitative real-time PCR

The expression of target genes was analyzed by quantitative Real-time PCR (qRT-PCR). qRT-PCR was performed on a CFX-96 qPCR thermal cycle instrument (Bio-Rad) and was carried out in a total volume of 10 μl with 1.0 μl complementary DNA preparations, 0.3 μl of each specific primer, 5 μl Ssofast EvaGreen (Bio-Rad) and 3.4 μl ddH_2_O. The optimum thermal cycling conditions were as follows: 98°C for 2 min, 39 cycles of 98°C for 10 s, 30 s at optimum temperatures, 72°C for 10 s, and a final extension for 5 min with a temperature increment of 0.5°C/s from 65°C to 95°C. The specificity of the qRT-PCR products were confirmed via melting curve analysis and 1.5% agarose gel analysis. The ALV-J genome load in the ALV-J-infected chicken tissues was quantified using primers specific for the ALV-J Env gene. The relative expression levels were calculated by the comparative Ct (2^-ΔΔCt^) method using glyceraldehyde-3-phosphate-dehydrogenase (GAPDH) gene as an endogenous reference gene. The gene-specific primers used for qRT-PCR of ALV-J *Env*, *GAPDH*, *chTBK1*, *IRF3* and *IFNβ* were listed in Table 1.

### Bioinformatics and statistical analysis

The obtained nucleotide sequences were analyzed at NCBI (http://blast.ncbi.nlm.nih.gov/) and were compared against the sequence database using the BLAST server (http://www.ncbi.nlm.nih.gov/blast). The conserved domain prediction was analyzed by the SMART program (http://smart.emblheidelberg.de/). Multiple amino acid sequences alignment was performed using ClustalX 1.83. The secondary structure was predicted by the SOPMA software (http://npsa-pbil.ibcp.fr/). The phylogenetic tree was constructed using the MEGA 5.05 software. The GenBank accession numbers of TBK1 from different species for the phylogenetic analysis were listed in Table 2.

**Table 2:** GenBank accession numbers of TBK1 homologs used in this study.

Name of species	Accession number
*Gallus gallus*	NP_001186487.1
*Meleagris gallopavo*	XP_003202091.1
*Taeniopygia guttata*	XP_002188051.2
*Columba livia*	XP_005499866.1
*Melopaittaous undulatus*	XP_005148126.1
*Oryctolagus cunioulus*	XP_008254959.1
*Homo sapiens*	NP_037386.1
*Ovis aries*	XP_004006546.1
*Capra hircus*	XP_005680278.1
*Bos taurus*	NP_001179684.1
*Sus scrofa*	NP_001098762.1
*Mus musoulus*	NP_062760.3
*Ornithorhynchus anatinus*	XP_007666451.1
*Danio rerio*	NP_001038213.2

The relative gene expression levels were calculated relative to the expression of the GAPDH gene, which served as an endogenous reference gene. The statistical analysis was performed using SAS 9.0 through one-way ANOVA. The means ± SEM results were plotted to the figures using GraphPad Prism 5 software (GraphPad Software, La Jolla, CA, USA). The comparisons were considered significant at *P<0*.*05* and extremely significant at *P<0*.*01*.

## Results

### Bioinformatic analysis of the chicken TBK1 gene

**Fig 1:**
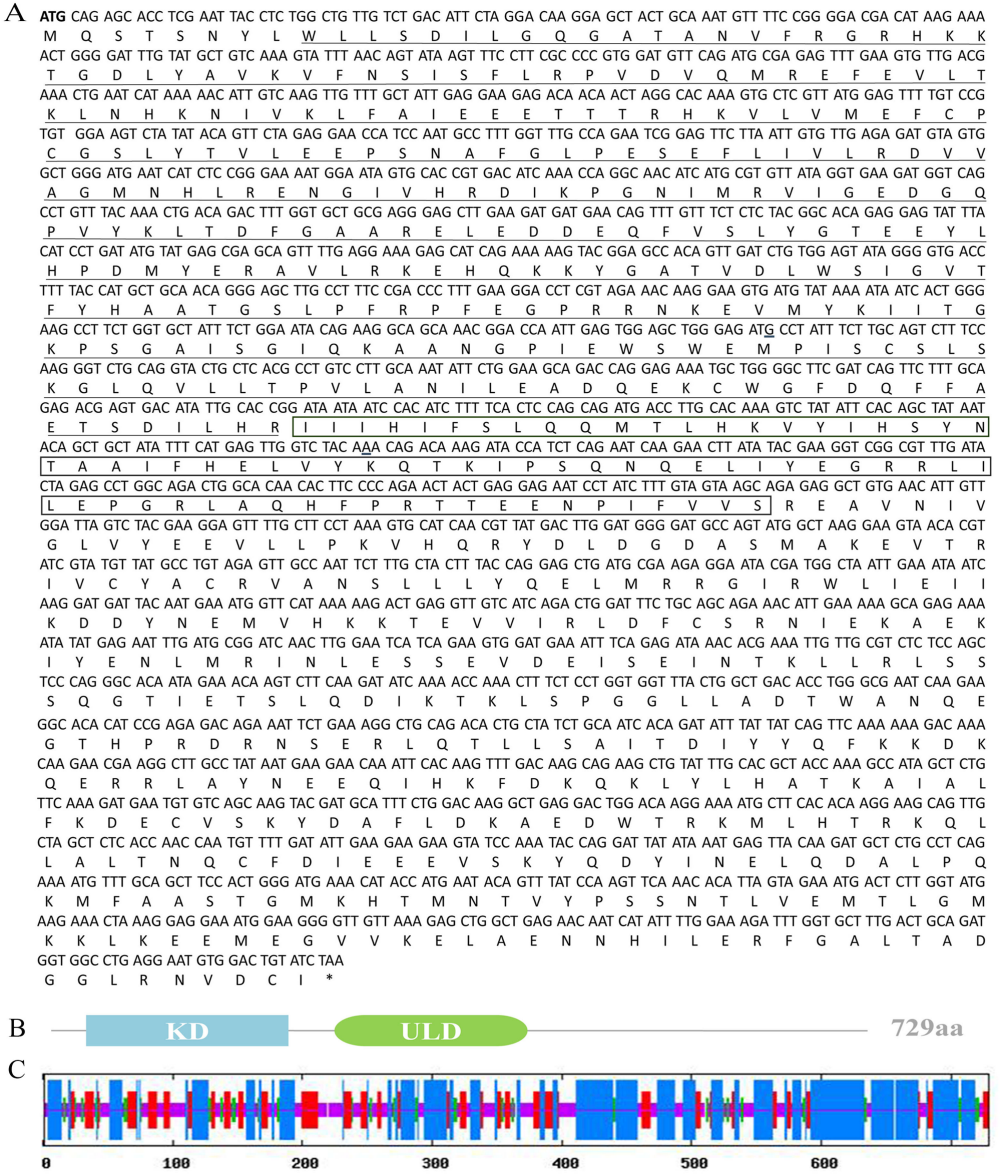
The *chTBK1* ORF sequence, deduced amino acid sequence and the second structure of chTBK1. **A**. The translation start (ATG) codon is in bold and the stop (TGA) codon is markerd by asterisis (*), the protein kinase domain is underlined and the ubiquitin-like domain is boxed. **B**. Schematic conserved domain structure of TBK1 protein, showing the protein kinase domain (KD) and ubiquitin-like domain (ULD). **C**. The longest lines, second longest lines, third longest lines and shortest lines stand for Alpha helixs (Hh), extended strands (Ee), beta turns (Tt), and random coils (Cc), respectively.

**Fig 2:**
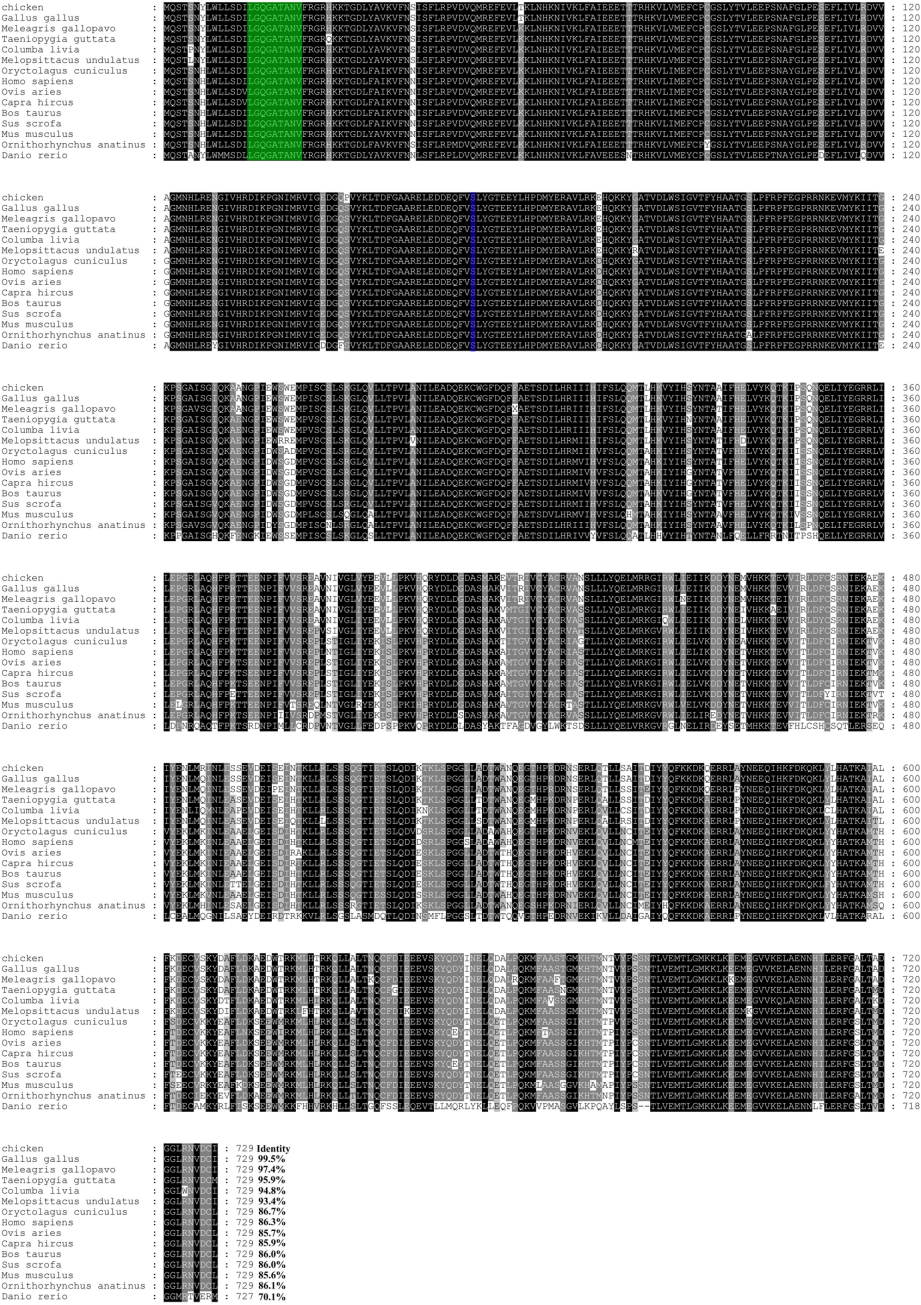
Multiple sequence alignment of TBK1 protein sequence of chicken and other 14 species by the ClustalW program. Residues shaded in black are completely conserved across all species aligned, and residues shaded in grey refer to 80–90% identity. The ATP binding site is in green, the S172 is in blue.

**Fig 3:**
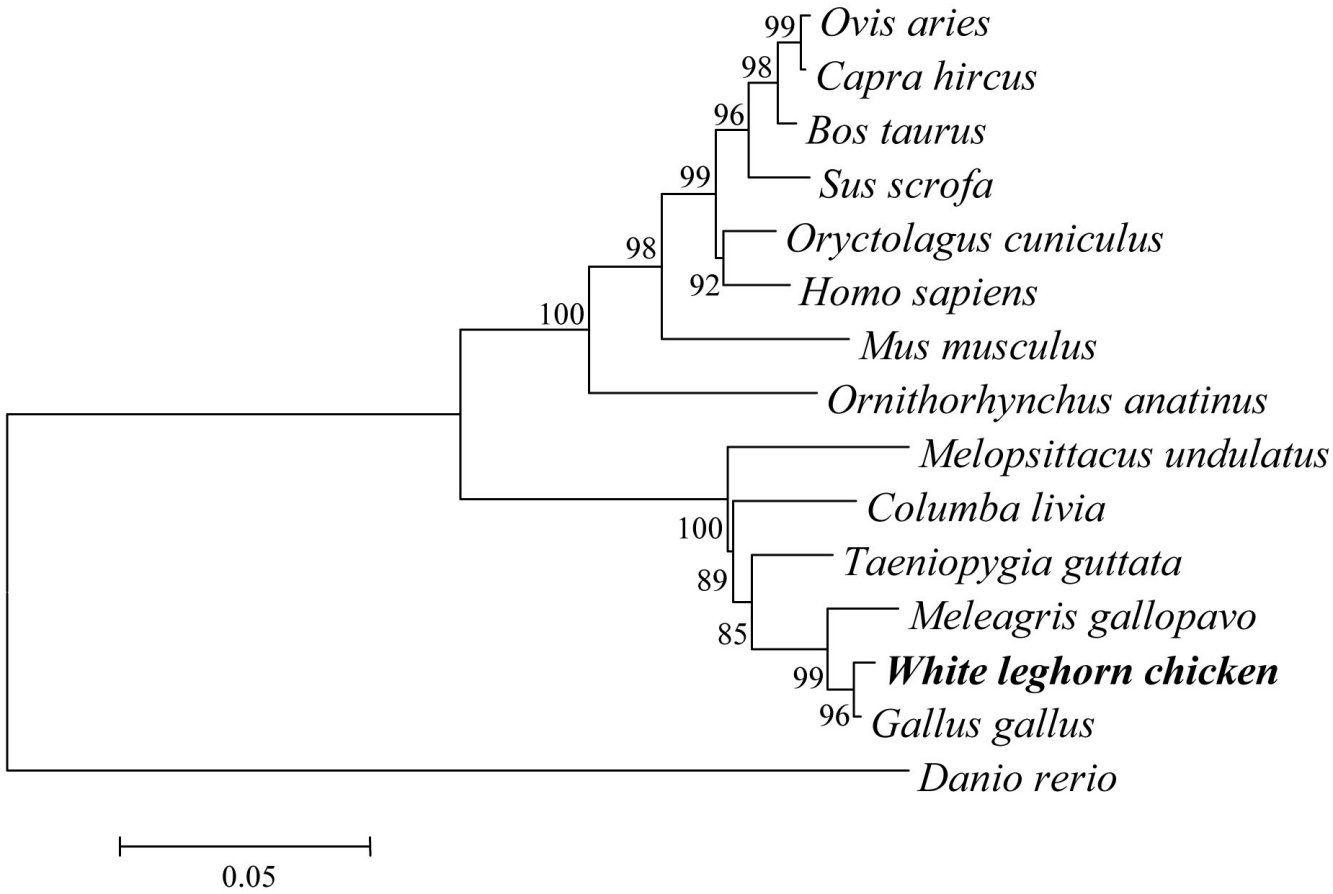
Phylogenetic trees analysis of chTBK1 proteins from different species. The phylogram was constructed with the MEGA 5.05 software using the neighbor-joining method based on an amino acid alignment (Clustal W) of the full-length protein. The scale bar indicates the branch length, and the bootstrap confidence values were shown at the nodes of the tree.

We successfully cloned and sequenced the full-length coding sequence of *chTBK1* based on the predicted *TBK1* sequence of *Gallus gallus* (GenBank accession number: NM_001199558.1). The full-length coding sequence of *chTBK1* was 2190 bp encoding a putative protein of 729 amino acids residues (Fig 1A). Based on NCBI CD-Search and SMART software analysis, two conserved domains including kinase domain and ubiquitin-like domain were predicted (Fig 1B). The secondary structure analysis using SOPMA software indicated that chicken TBK1 was comprised of 47.19% α-helixes, 26.47% random coils connected by 19.07% extended strands and 7.27% β-turns (Fig 1C). The multiple alignments revealed that chTBK1 shares the highest identity (99.5%) with *Gallus gallus* followed by *Meleagris gallopavo* (97.4%), *Teaniopygia gttata* (95.9%), *Columba livia* (94.8%), *Melopaittaous undulatus* (93.4%), *Oryctolagus cunioulus* (86.7%), *Homo sapiens* (86.3%), *Ovis aries* (85.7%), *Capre hirous* (85.9%), *Bos taurus* (86.0%), *Sus scrofa* (86.0%), *Mus musoulus* (85.6%), *Ornithorhynous anatinus* (86.1%) and displayed relatively low identity with *Danio rerio* (70.1%) (Fig 2). As observed in Fig 2, the ATP-binding site and S172 residue among the kinase domain of TBK1 were identical. To elucidate the genetic relationships of the chicken TBK1 protein with other species, we constructed a phylogenetic tree using MEGA 5.0 software with the neighbor joining (NJ) method and 1000 replicates (Fig 3). The TBK1 amino acid sequence from mammals, birds and fish segregated into 3 separate clusters, and the chicken TBK1 was grouped into the birds cluster containing *Meleagris gallopavo*, *Teaniopygia gttata*, *Columba livia* and *Melopaittaous undulatus* but was phylogenetically separated from that of other mammalian species. The phylogenetic tree analysis suggested that chTBK1 had a closer genetic relationship with that of other birds in comparison to other vertebrates. These results were consistent with previous studies.

### Tissue distribution of chTBK1 mRNA in healthy chicken

**Fig 4:**
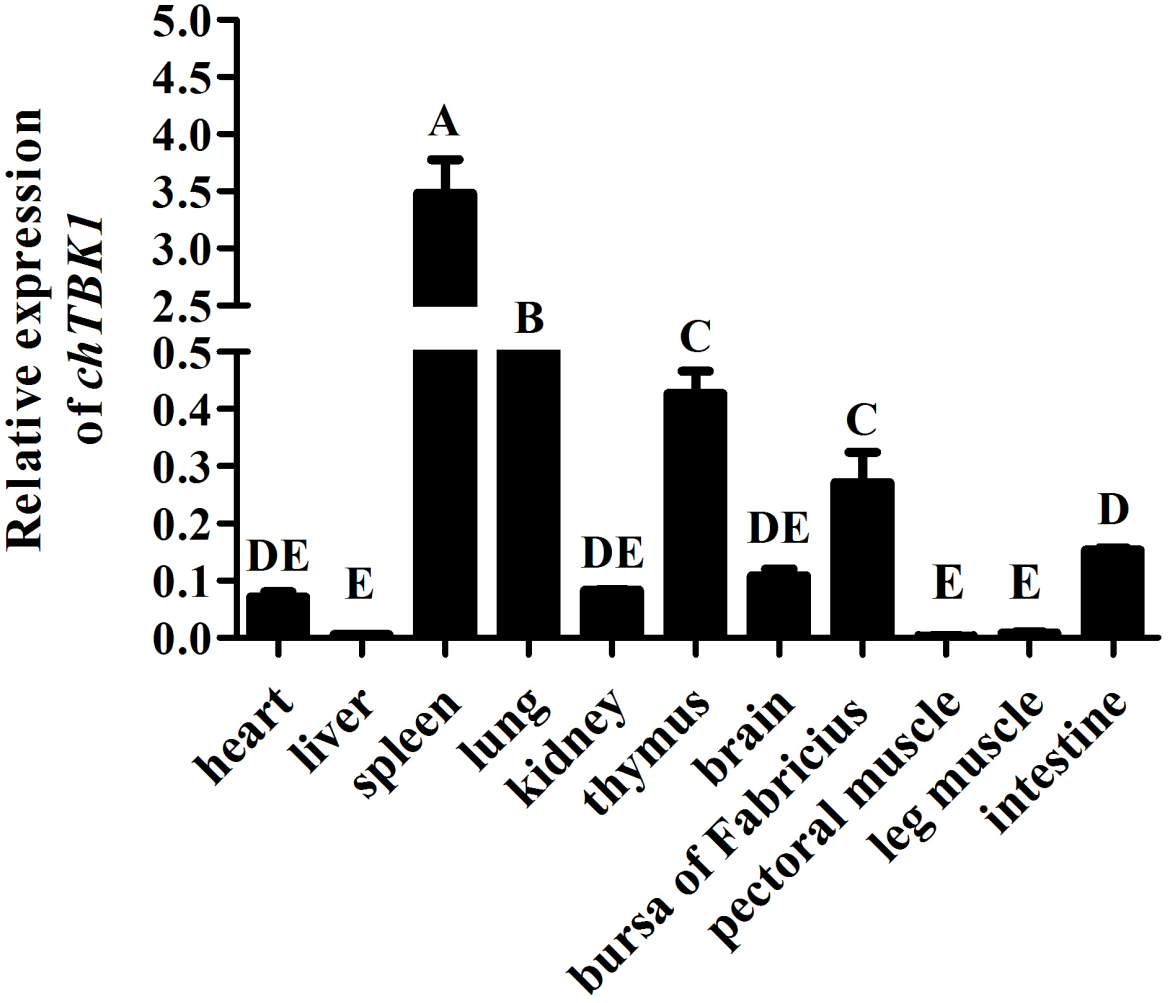
Relative mRNA expression of chTBK1 gene in different tissues, including heart, liver, spleen, lung, kidney, thymus, brain, bursa of Fabricius, pectoral muscle, leg muscle, intestine. Data above were presented as mean ± SEM (n = 3). Error bars show the SEM of triplicate. Columns sharing different letters show extremely significant difference (*P<0*.*01*).

To assess the mRNA expression profile of the chTBK1 gene, 11 tissues were collected, and the transcript levels of target gene were compared using the GAPDH gene as the internal reference gene for normalization. As shown in Fig 4, the chTBK1 gene was ubiquitously expressed in all of the examined tissues. The most abundant *chTBK1* expression levels were found in spleen, lung, thymus, bursa of Fabricius, which was followed by intestine, heart, kidney and brain. Low expression levels were found in the other tissues, including liver, pectoral muscle and leg muscle. The statistically analysis revealed that the transcripts level of *chTBK1* in spleen, lung, thymus, bursa of Fabricius were significantly (*P<0*.*01*) higher than those in the other 7 tissue types.

### Response of chTBK1 in response to ALV-J infection in vivo

**Fig 5:**
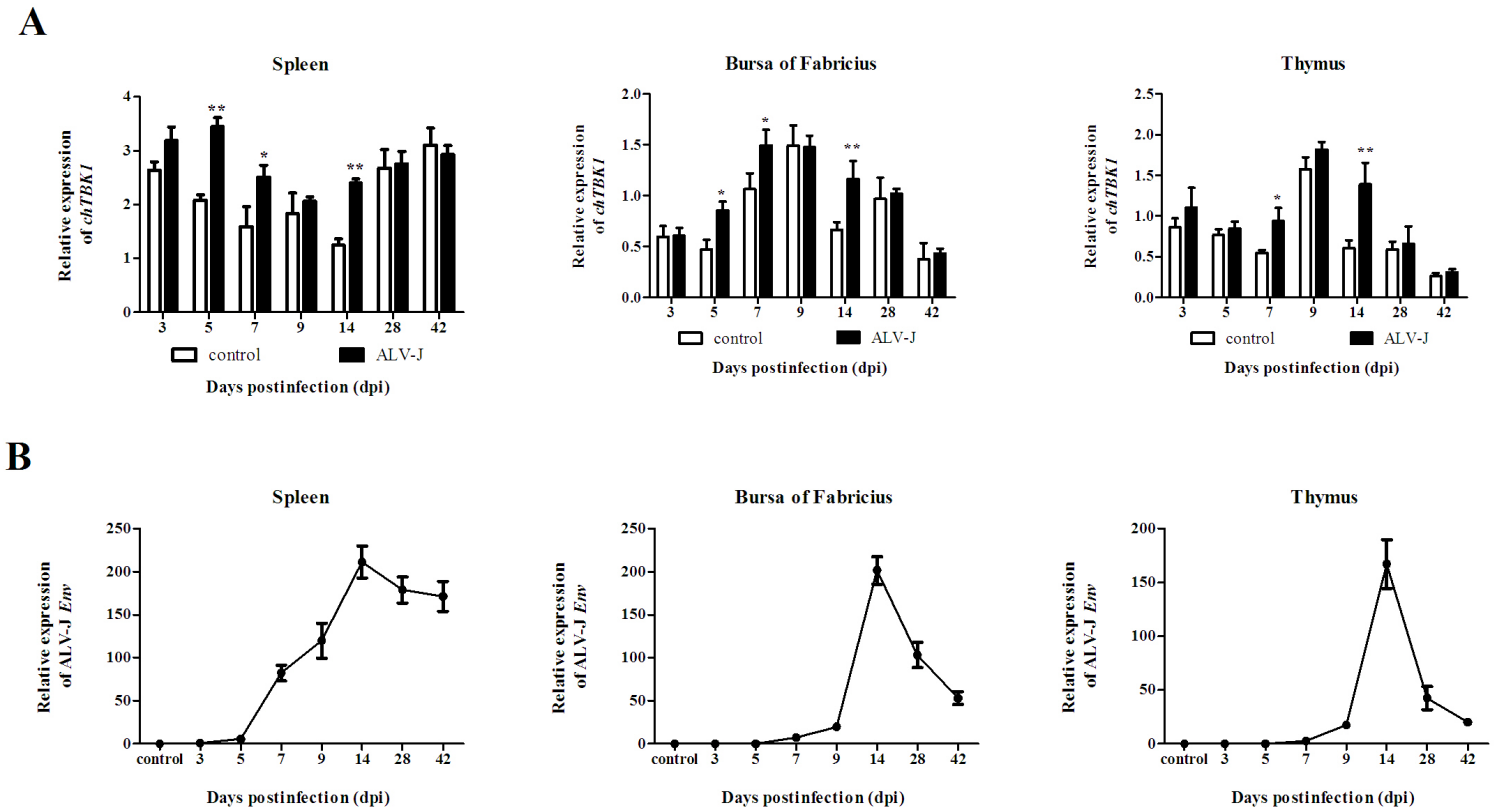
Relative mRNA expression of ALV-J Env and chTBK1 gene in vitro challenge with ALV-J in different immune tissues. **A** Relative mRNA expression of chTBK1 gene in the spleen, bursa, and thymus after challenge with ALV-J. Data above were presented as mean ± SEM (n = 3). Error bars show the SEM of triplicate. (*) and (**) represent significant (*P < 0*.*05*) and extremely significant (*P <0*.*01*) difference, respectively. **B** Relative mRNA expression of ALV-J Env gene in the spleen, bursa, and thymus of ALV-J infected chickens.

To determine if *chTBK1* is involved in chicken immune responses, the expression level of *ch*TBK1 and the ALV-J Env gene at different time points following infection with ALV-J in spleen, bursa of Fabricius and thymus were determined by qRT-PCR. As shown in Fig 5A, the expression of *chTBK1* was up-regulated after ALV-J infection when compared to the non-infected group. In the spleen, *chTBK1* was activated at 5 (*P<0*.*01*), 7 (*P<0*.*05*) and 14 (*P<0*.*01*) dpi. In the bursa of Fabricius, a similar variation trend was found, but *chTBK1* was increased at 5(*P<0*.*05*), 7 (*P<0*.*05*) and 14 (*P<0*.*01*) dpi. In the thymus, *chTBK1* was up-regulated only at 7 (*P<0*.*05*) and 14 (*P<0*.*01*) dpi. As shown in Fig 5B, the ALV-J Env gene was quantifiable as early as 5 dpi in the spleen, and was detected later in the bursa of Fabricius. In the spleen, the expression of the ALV-J Env gene increased quickly, peaked at 14 dpi and maintained a higher expression level at 28 and 42 dpi. In the bursa of Fabricius and thymus, the expression of the ALV-J Env gene peaked at 14 dpi but phased down immediately.

### Induction of chTBK1, IRF3 and IFNβ after in vitro challenge with ALV-J

**Fig 6:**
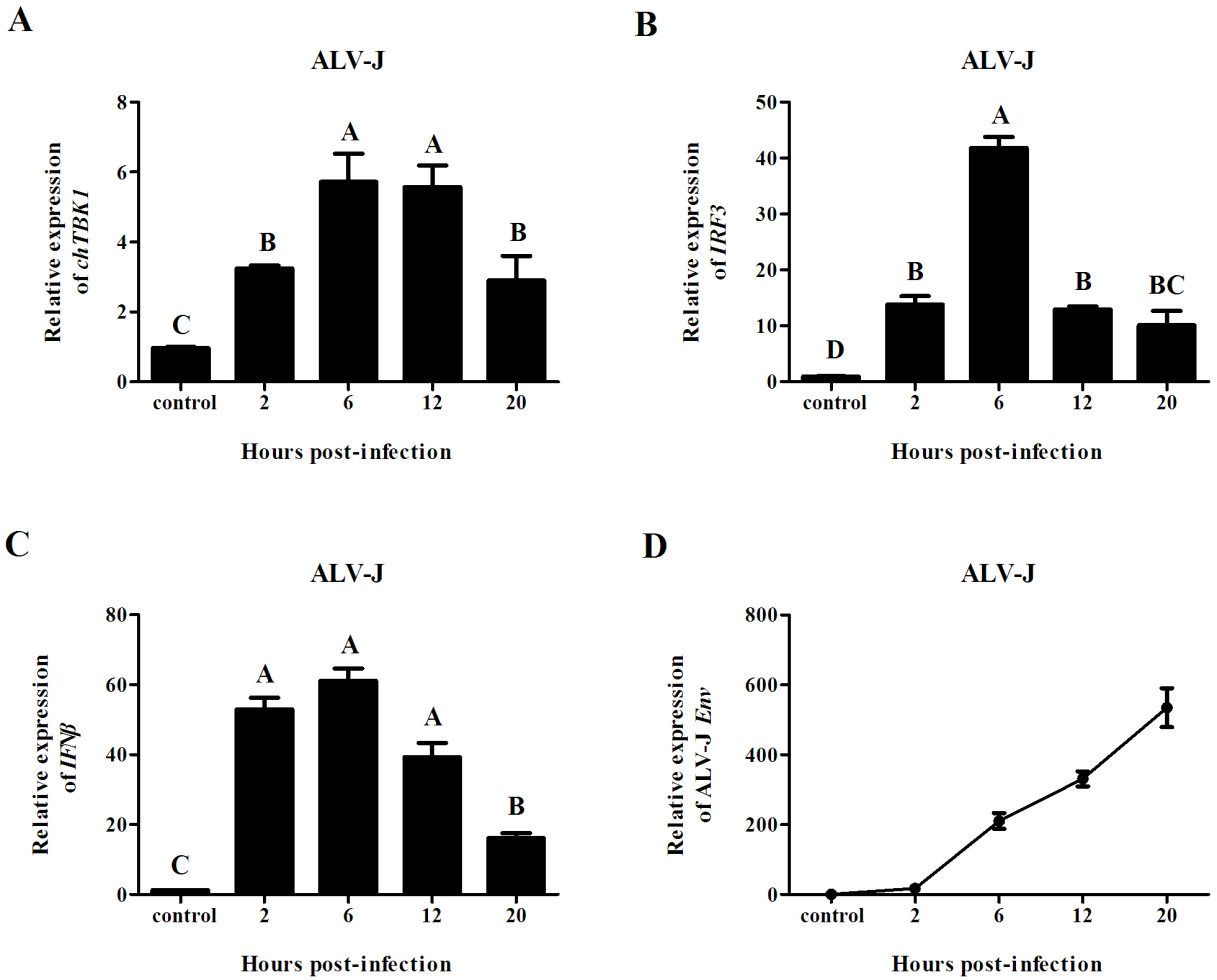
Relative mRNA expression of chTBK1, IRF3, IFNβ and ALV-J Env gene in CEFs challenge with ALV-J, showing in A, B, C and D, respectively. Data above were presented as mean ± SEM (n = 3). Error bars show the SEM of triplicate. Columns sharing different letters show extremely significant difference (*P<0*.*01*).

To further characterize the role of the *chTBK1*, we next investigated the potential role of *chTBK1* in cellular antiviral responses. As only TBK1 (not IKKε) was found in mouse embryonic fibroblasts by Northern blotting analysis, we choose CEFs as the cell model. After challenge with ALV-J, the chTBK1 gene sharply increased from 2 hpi and peaked at 6 and 12 hpi (Fig 6). Concurrently, the expression levels of *IRF3* and *IFNβ* peaked at 6 hpi and then gradually decreased. Meanwhile, the ALV-J Env gene increased exponentially at 2, 6, 12 and 20 hpi.

### Induction of chTBK1, IRF3 and IFNβ after in vitro challenge with polyI:C

**Fig 7:**
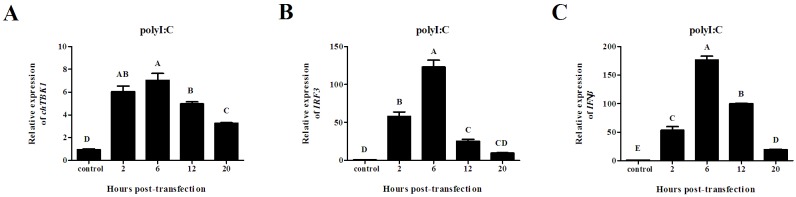
Relative mRNA expression of chTBK1, IRF3 and IFNβ gene in CEFs transfection with polyI:C, showing in A, B and C, respectively. Data above were presented as mean ± SEM (n = 3). Error bars show the SEM of triplicate. Columns sharing different letters show extremely significant difference (*P<0*.*01*).

The dsRNA generated during the virus replication could rapidly trigger host innate immune responses. To explore whether chTBK1 was implicated in chicken cell responses to dsRNA stimulation, the CEFs were transfected with polyI:C (a synthetic dsRNA) and were harvested at different time points. As shown in Fig 7, following transfection with 1.0 μg/ml polyI:C the transcription level of *chTBK1*, *IRF3* and *IFNβ* in CEFs gradually increased from 2 hours and peaked at 6 hours.

### ChTBK1 knockdown blocks IRF3 as well as IFNβ activation

**Fig 8:**
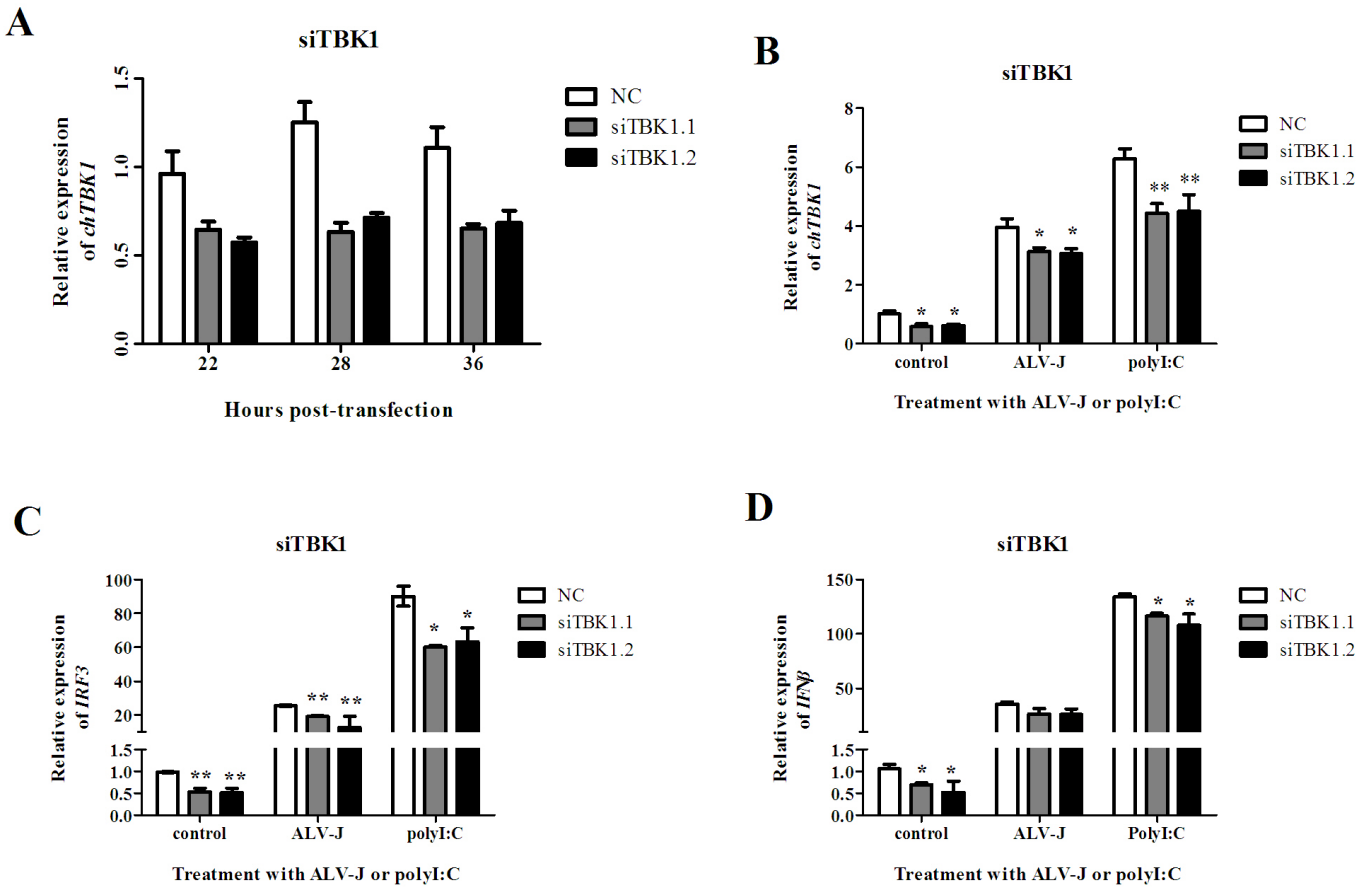
Knockdown of chTBK1 reduced IRF3 and IFNβ gene expression in CEFs. **A** Effects of siTBK1 on the expression of endogenous *chTBK1* in CEFs at different time. **B, C, D** Relative mRNA expression of chTBK1, IRF3 and IFNβ gene in chTBK1 knockdown CEFs challenge with ALV-J as well as polyI:C, compared to negative control (NC), respectively. Data above were presented as mean ± SEM (n = 3). Error bars show the SEM of triplicate. (*) and (**) represent significant (*P < 0*.*05*) and extremely significant (*P <0*.*01*) difference, respectively.

To further understand the function of chTBK1, *chTBK1* was knocked down in the CEFs. Two small interfering RNAs (siRNA) targeting different portions of *chTBK1* (siTBK1.1, siTBK1.2) and a negative control (NC) siRNA were prepared. Before chTBK1 knock-down in CEFs challenged with ALV-J or polyI:C, a pilot experiment evaluated the effectiveness of siTBK1.1 and siTBK1.2. As shown in Fig 8A, both *chTBK1* siRNAs led to a 40–50% reduction of *chTBK1* transcription level in CEFs at 22–36 hours post-transfection compared to the parallel control. We the challenged CEFs with ALV-J and poly I:C 24 hours after transfection with siTBK1, and 4 hours later all the cells were collected. As shown in Fig 8, the expression of *chTBK1*, *IRF3* and *IFNβ* was determined by qRT-PCR. As expected, the expression of *chTBK1* sharply decreased in CEFs transfected with siTBK1.1 and siTBK1.2 compared to the NC, regardless of whether the CEFs were not challenged or were challenged with ALV-J or polyI:C (Fig 8B). Interestingly, the transcription level of *IRF3* and *IFNβ* were significantly reduced in *chTBK1* knock-down CEFs challenged with ALV-J or polyI:C, as shown in Fig 8C and 8D, respectively.

## Discussion

TBK1 is a critical adaptor molecule that is involved in the TLRs, RLRs and STING signaling pathways that induce type I IFN production after virus infection in mammals. To date, TBK1 has been well-characterized in a number of mammalian species and teleosts [20], but few studies have been conducted in avian species. In this study, we cloned and characterized the chTBK1 gene. The full-length coding sequence *chTBK1* consisted of 2190bp encoding for 729 amino acid residues. While chTBK1 clusters in the avian group, multiple sequence alignment analysis showed that chTBK1 has high homology with TBK1 of mammals, ranging from 85.6% to 97.4%. Conserved domains were identified in the N-terminus of the chTBK1 protein, including the protein kinase domain and the ubiquitin-like domain, which is identical with murine and human TBK1 [5,6]. Of note, the ATP-binding site (LGQGATANV) and Ser172 residue, which are located in the protein kinase domain, were completely conserved in all of the TBK1 kinases and is consistent with those found in other species [20]. The mammalian TBK1 crystal structure showed that the kinase domain of TBK1, which harbors an ATP-binding site and a phosphorylation site (Ser172), is a structurally conserved protein domain containing the catalytic function of this protein kinase [6]. The ubiquitin-like domain functions as a protein-protein interaction domain, which is implicated in regulating kinase activity, substrate presentation and downstream signaling pathways [7]. Our results showed that chTBK1 is very similar to the human and murine orthologs in terms of the sequence and structure. The similarity is especially high in the conserved domain, which suggests that chTBK1 may have a similar function to the human and murine TBK1.

The expression of *TBK1* has been investigated in many vertebrate species. In contrast to its closest ortholog *IKKε*, *TBK1* is ubiquitously expressed in most tissues and cell types [21]. In agreement with those observations, our results demonstrated that *chTBK1* was differentially expressed in all of the examined tissues. Of note, it was highly expressed in the spleen, thymus, and lung, which are all immune-related tissues, suggesting that chTBK1 may be an important factor in the immune system of chickens.

ALV-J is a single-strand RNA virus belonging to the retroviridae. Similarly to human immunodeficiency virus, ALV-J replication cycle involves in provirus integration and the synthesis of viral proteins. During this cycle, the genomic information is carried in different forms, including single-strand RNA, RNA:DNA hybrid, single-strand DNA, dsDNA and dsRNA [22,23]. Previous studies in other retroviruses demonstrated that single-strand RNAs were recognized by the endosomes of specialized innate immune cells using TLR7, while reverse-transcribed DNA is believed to activate STING signaling pathways by cytoplasmic DNA sensors, such as cyclic GMP-AMP synthase and IFI16 [11,24,25]. Additionally, according to Liu et al, ALV-J is mainly recognized by the RIG-I-like signaling pathways in chicken [26]. Although RIG-I is absent in chickens, MDA5 (the closest ortholog of RIG-I), TLR7 and STING have been identified. Moreover, previous studies indicated that chMDA5 can functionally compensate for the absence of RIG-I [27]. TBK1 is thought to be a common adaptor molecular in TLR, RLR and STING signaling pathways. Recently, a number of studies revealed that TBK1 is activated by various viruses, such as adenovirus [28], hepatitis C virus [29] as well as retrovirus like human immunodeficiency virus [25]. In this study, the expression of *chTBK1* in the spleen was significantly up-regulated (*P<0*.*01*) as early as 5 dpi when the ALV-J Env gene was first detected. These results further confirmed that the spleen is a lymphoid organ that is reached early by the virus and is involved in the early innate host responses [30]. Moreover, the transcription of *chTBK1* was significantly up-regulated in all of the detected immune tissues at 14 dpi when a large amount of ALV-J is distributed in the three organs, which indicated there was a strong correlation between ALV-J copy number and innate immune responses. These findings are consistent with observations of Newcastle disease virus and avian influenza virus infection [31]. In summary, increased *chTBK1* expression was observed in chickens after infection with ALV-J in the three main immune organs compared to the control, which suggests a potential antiviral role for chTBK1 in chickens.

Because TBK1 was constitutively expression in almost all cell types, whereas IKKε was mainly found in immune cells [17], embryo fibroblasts are considered to be a good cell model to investigate TBK1. Previous studies reported that embryo fibroblasts primarily detect virus infection through cytoplasmic receptors called RLRs [32]. In chickens, MDA-5 has been investigated and is involved in sensing dsRNA and influenza A virus [27,33,34]. As it is known that TBK1 is essential for the IFN responses against viruses or dsRNA recognized by both RIG-I and MDA5 [35], we speculated that *chTBK1* would be up-regulate in CEFs after ALV-J or polyI:C challenge. As expected, the expression of *chTBK1* was significantly increased at 2 hpi and peaked at 6 and 12 hpi in CEFs after ALV-J infection, suggesting that ALV-J could initiate the innate immune response of host cells as was found in the in vivo study. In addition, increases in the expression levels of the chTBK1 gene was also observed at 2 hours and peaked at 6 hours in CEFs transfected with polyI:C, suggesting the involvement of TBK1 in immune responses triggered by dsRNA in chicken cells. These results corroborated the in vivo data and were the first evidence to suggest that chTBK1 is involved in anti-virus immune responses both *in vivo* and *in vitro*.

As mentioned above, chTBK1 is essential for anti-virus immune responses in chickens. In mammals, TBK1 was identified as a main kinase that mediates type I interferon expression by phosphorylation of IRF3/IRF7, especially in fibroblasts [17]. Surprisingly, in chickens, only IRF3 was found [36–38]. Moreover, chicken IRF3, which is closely related to mammalian IRF7 in structure, may induce IFNβ expression after virus or dsRNA stimulation [37,39]. This information suggestes that the *ch*TBK1 gene may induce *chIRF3* and *IFNβ* expression. As expected, significant increases in the expression levels of chicken IRF3 and IFNβ gene were observed in CEFs challenge with ALV-J and polyI:C, which is in agreement with the findings of previous studies [27,37]. We also observed that the chicken IRF3 and IFNβ genes have similar expression level changes to the *ch*TBK1 gene, which implied that the expression level of chicken IRF3 and IFNβ correlates with the activation of chTBK1.

To address this potential correlation, we studied the effects of chicken TBK1 knockdown after challenge with ALV-J and polyI:C. After transfection with siTBK1.1 and siTBK1.2, the expression of *chTBK1* was significantly reduced (40%-50%). Simultaneously, both *IRF3* and *IFNβ* were significantly impaired after ALV-J and polyI:C treatment in chTBK1-deficient CEFs compared to the NC, which is consistent with the analysis of *IRF3* and *IFNβ* expression in both TBK1- deficient cells and mice [15, 16]. These data further confirmed that chTBK1 induces *IRF3* and *IFNβ* transcription in chicken cells. Previous studies have shown that upon activation TBK1 directly phosphorylates the C-terminal domain of IRF3 [16]. Phosphorylation ultimately results in IRF3 dimerization and translocation into the nucleus where the IRF3 homodimers cooperate with CREB-binding protein (CBP)/P300 to induce the expression of the type I interferon gene [40,41]. IFNβ is the main type I interferon group member. Thus, these previous results and our present observations suggest that chTBK1 likely functions as an adaptor molecule to resist a virus-induced antiviral signaling pathway in chicken cells. Further functional studies are required for determining the exact role of chTBK1 in antiviral immune responses. These results will help us to further clarify the molecular mechanisms of chicken antiviral immune responses.

In conclusion, we have conducted comprehensive structural characterizations and immune function analysis of chTBK1. The ORF of *chTBK1* consists of 2190 bp encoding 729 amino acid residues. The putative chTBK1 protein is structurally and phylogenetically closely related to human and mouse TBK. *ChTBK1* is highly expressed in immune or other immune-related tissues, including spleen, thymus, bursa of Fabricius and lungs. Furthermore, *chTBK1* was significantly increase *in vivo* or *in vitro* challenge with ALV-J and polyI:C, respectively. Our results suggested that *chTBK1* has an important role in virus infection or dsRNA stimulation in innate immunity. In addition, augmentation of *chTBK1* resulted in *IRF3* and *IFNβ* up-regulation *in vitro* after ALV-J infection or polyI:C transfection. Conversely, *IRF3* and *IFNβ* induction with reduced in TBK1-defective CEFs. Collectively, these results demonstrate that chTBK1 is required for type I interferon induction against viral infection and dsRNA stimulation. Importantly, IRF3 likely functions as a main adaptor molecular of chTBK1 signaling, which is similar to the role of mammalian IRF3.
